# Lost region in amyloid precursor protein (APP) through TALEN-mediated genome editing alters mitochondrial morphology

**DOI:** 10.1038/srep22244

**Published:** 2016-02-29

**Authors:** Yajie Wang, Fengyi Wu, Haining Pan, Wenzhong Zheng, Chi Feng, Yunfu Wang, Zixin Deng, Lianrong Wang, Jie Luo, Shi Chen

**Affiliations:** 1 Key Laboratory of Combinatorial Biosynthesis and Drug Discovery, Ministry of Education, School of Pharmaceutical Sciences, and Medical Research Institute, Wuhan University, Wuhan 430071, China; 2 Taihe Hospital, Hubei University of Medicine, Shiyan, Hubei, China

## Abstract

Alzheimer’s disease (AD) is characterized by amyloid-β (Aβ) deposition in the brain. Aβ plaques are produced through sequential β/γ cleavage of amyloid precursor protein (APP), of which there are three main APP isoforms: APP_695_, APP_751_ and APP_770._ KPI-APPs (APP_751_ and APP_770_) are known to be elevated in AD, but the reason remains unclear. Transcription activator-like (TAL) effector nucleases (TALENs) induce mutations with high efficiency at specific genomic loci, and it is thus possible to knock out specific regions using TALENs. In this study, we designed and expressed TALENs specific for the C-terminus of APP in HeLa cells, in which KPI-APPs are predominantly expressed. The KPI-APP mutants lack a 12-aa region that encompasses a 5-aa trans-membrane (TM) region and 7-aa juxta-membrane (JM) region. The mutated KPI-APPs exhibited decreased mitochondrial localization. In addition, mitochondrial morphology was altered, resulting in an increase in spherical mitochondria in the mutant cells through the disruption of the balance between fission and fusion. Mitochondrial dysfunction, including decreased ATP levels, disrupted mitochondrial membrane potential, increased ROS generation and impaired mitochondrial dehydrogenase activity, was also found. These results suggest that specific regions of KPI-APPs are important for mitochondrial localization and function.

Alzheimer’s disease (AD) is a neurodegenerative disorder typically affecting individuals over 65 years of age. The main pathological characteristic is the deposition of senile plaques, β-amyloid (Aβ) plaques produced via amyloid precursor protein (APP) processing, in the brain. APP is a type I trans-membrane protein^1^. Newly synthesized APP is transported from the Golgi to the cell surface and cleaved by α/γ secretase in the “non-amylogenic pathway”^2^. When cell membrane-localized APP is reinternalized into the endosomal compartment containing β and γ secretases, Aβ is generated, which may then be degraded in the lysosome or transported into the extracellular space^3^. The trans-membrane region (TM) of APP is critical for processing and subcellular localization^4^. There are three main isoforms of APP (APP_695_, APP_751_, and APP_770_) produced through alternative splicing of exons 7 and 8. APP_695_ is primarily expressed in neurons^5^. The APP_751_ isoform contains an additional Kunitz-type protease inhibitor (KPI) domain, and APP_770_ an extra OX-2 domain^6^; both are expressed rather ubiquitously^7^. Expression of APP_751_ and APP_770_ is elevated in the brain of AD patients^5^,though the pathology remains unknown.

Although mitochondrial dysfunction is known to be an early event in AD^8^, knowledge regarding APP function in the mitochondria is limited. In mouse cortical neuronal cells, APP_695_ is targeted to the mitochondria with its N-terminus oriented toward the inside of the organelle^9^. In the AD vulnerable region, frontal cortex, hippocampus and amygdala, APP_695_ or Aβ is present in the mitochondrial membrane, blocking mitochondrial import channels and impairing mitochondrial function by disrupting protein localization and the electron transfer chain and, resulting in mitochondrial damage^10^. The overexpression of both APP_695_ and APPs we causes alterations in mitochondrial morphology and distribution, suggesting a role for these proteins in mitochondrial dynamics and function^11^. However, the mechanism by which KPI-containing APPs, APP_751_ and APP_770_ influence mitochondrial function remains elusive.

Mitochondrial dysfunction plays a role in the pathogenesis of AD^12^. Mitochondria undergo continuous cycles of fission and fusion; the key fusion mediators include Mitofusin 1 (MFN1)^13^, Mitofusin 2 (MFN2)^14^ and optic atrophy 1 (OPA1)^15^, whereas dynamin-related protein 1 (DRP1)^16^ regulates fission. An imbalance in fusion and/or fission leads to changes in mitochondrial morphology, which could represent an important mechanism underlying mitochondrial dysfunction in the AD brain^17^. In APP_695_- and APP_swe_- overexpressing human neuroblastoma cells, DRP1 and OPA1 are significantly decreased, along with fragmented and punctiform mitochondria^11^, and overexpression of APP_751_ significantly decreases the expression levels of mitochondrial metabolic enzymes as well as the mitochondrial membrane potential^18^. However, these results are based on ectopic overexpression, and the observations might be artifactual. As transcription activator-like effector nucleases (TALENs) are useful tools for genome editing and knocking out specific domains, we generated a 36-bp *app* deletion in HeLa cells using TALENs. Both KPI-APP isoforms, APP_751_ and APP_770_, lack a 12-aa region covering a 5-aa trans-membrane region (TM) and a 7-aa juxta membrane region (JM). Mutated KPI-APPs show decreased mitochondrial localization, and altered mitochondrial morphology in cells expressing these mutants occurs through disruption of the fission and fusion balance. Therefore, loss of this region in KPI-APPs induces mitochondrial dysfunction.

## Results

### Generation of *app*- mutated cell lines through TALEN-mediated genome editing

**Figure 1:**
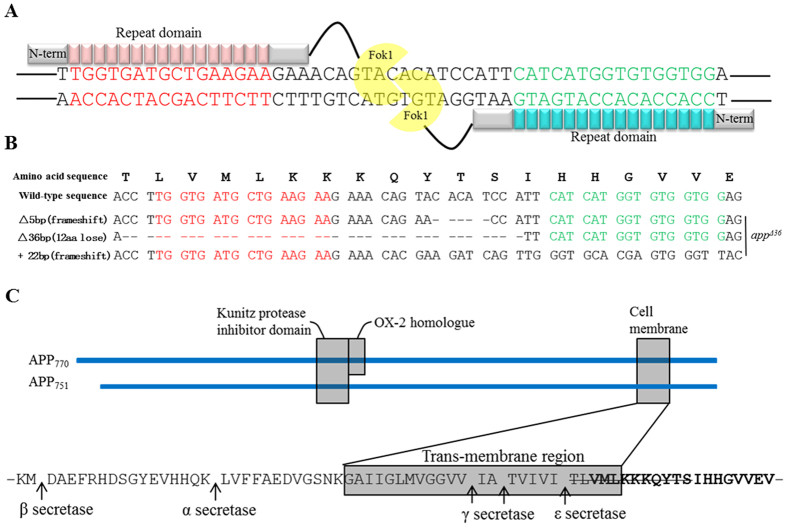
Generation of the TALEN-mediated *app^Δ36^*. (**A**) The DNA binding sequences (red or green) and spacer region (black) for TALENs are located in the 17 exon of APP_770_. (**B**) The cDNA sequences of the *app* locus from *app^Δ36^*; “-” denotes deleted nucleotides. (**C**) Sequence of deleted region in *app^Δ36^* (amino acids stricken-through). Sequence in the gray box represents TM region. Bolded sequence represents AICD region. The arrow indicates α, β, γ, and ε secretase sites.

To target a specific region of *app* in the genome, highly active TALENs (*app*-TALENs) specific to exon 17 of *app* were designed (Fig. 1A), and plasmids encoding TALEN binding pairs were transfected into HeLa cells. To estimate the efficiency of *app*-TALENs, genomic DNA from transfected cells was prepared after 48 h. The PCR-amplified products (1F/1R) covering exon 17 were sequenced, and multiple peaks at the target site suggested that the *app*-TALENs efficiently induced mutations. Single clonal mutants were isolated, expanded and selected by immunoblotting and sequencing of the T/A-cloned PCR products (1F/1R). Wild-type *app* was not detected in the cell line named *app^Δ36^*, and 3 types of mutations were found at *app* locus in this mutant: a 5-bp deletion (Δ5 bp), a 22-bp insertion (+22 bp) and a 36-bp deletion (Δ36 bp) (Fig. 1B). Both the 5-bp deletion and 22-bp insertion may lead to frame-shifts, constituting an *app* knockout. The 36-bp in-frame deletion results in the loss of a 12-aa region including the TM region (5-aa) and JM region (7-aa) at the C-terminus (Fig. 1C).

**Figure 2:**
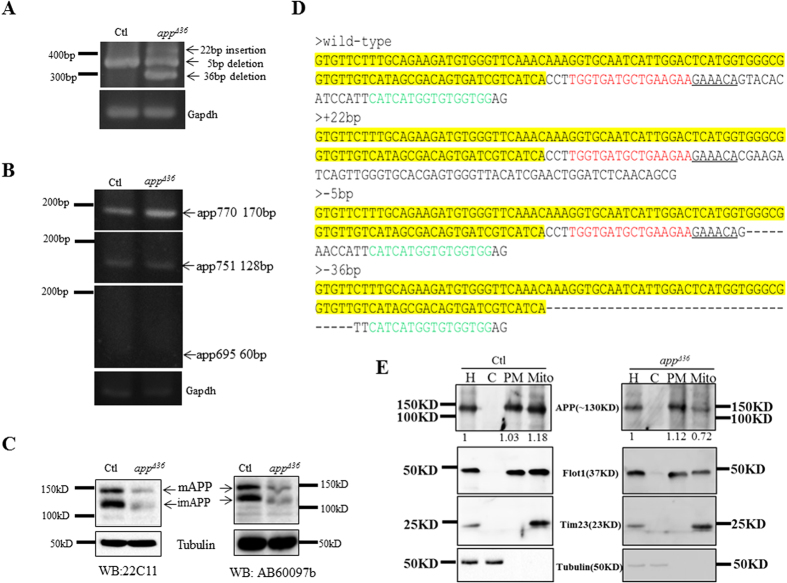
Mutations in the *app* locus of *app^Δ36^*. (**A**) PCR products of mutated *app* cDNA are of different lengths. (**B**) RT-PCR products corresponding to cDNA of the three APP isoforms on a 3% agarose gel. APP isoforms: APP_695_ (60 bp), APP_751_ (128 bp) and APP_770_ (174 bp). (**C**) Immunoblotting analysis for APP in *app^Δ36^* using two antibodies. The arrows indicate mature and immature APP in HeLa cells. (**D**) The cDNA sequences of the *app* locus from control and *app^Δ36^* cells. DNA binding sequences (red or green) for TALENs and the identical sequences (yellow) are indicated. “-” denotes deleted nucleotides. (**E**) Immunoblot analysis confirmed cellular localization of APP in *app^Δ36^* cells. The cell homogenate (H) was separated into cytoplasm (C), plasma membrane (PM) and mitochondria (Mito). The fractions were verified by Tubulin (C), Flotillin 1 (PM) and Tim23 (Mito). A 1% portion of the cell homogenate (H) was loaded as quantity control. PM and Mito from the 90% homogenate, and C from the 1% homogenate were loaded for analysis.

To confirm the TALEN-induced mutations, the levels of *app* mRNA and protein were then measured. Primers (2F/2R) were designed corresponding to *app* exon 16 and exon 18. The wild-type *app* mRNA RT-PCR product is 350 bp, but the mutation products were expected to be of various lengths. Three bands were detected on 3% gels (Fig. 2A). To verify the mutations, all bands were gel purified and sequenced after T/A cloning, and mutated cDNA with the 5-bp deletion (Δ5 bp), 22-bp insertion (+22 bp) and 36-bp deletion (Δ36 bp) were detected (Fig. 2D), whereas no wild-type *app* was found. We detected APP using two different antibodies (anti-22C11 and anti-AB60097b). Then weaker signal on APP immunoblots indicated that only APP lacking the 12-aa region was expressed in *app^Δ36^* cells (Fig. 2C).

To investigate the isoforms expressed in HeLa cells, we used primers that bound specifically to APP_770_ (APP7U and APP8L), APP_751_ (APP7U and APP7/9L) and APP_695_ (APP6/9U and APP9L) cDNA^19^. As expected, APP_695_ was not detected in HeLa cells, whereas both APP_751_ and APP_770_, containing the KPI domain, were detected (Fig. 2B). These results indicated that *app^Δ36^* KPI-APPs lack the 12-aa region.

### Mis-localization of APP in *app^Δ36^* cells

As the TM and JM regions are critical for APP processing^20^ and subcellular localization^4^, we expected that the loss of these regions in KPI-APPs might alter localization of the protein. Because the expression level of mutated KPI-APPs was lower than wild-type APP in control cells, we compared the relative proportion of APP in cellular fractions in control and *app^Δ36^* cells. The cell homogenate (H) was separated into the pellet (P1) and supernatant (S1), and a crude mitochondria preparation (Mito) in the P1 fraction was verified by the presence of Tim23 (Fig. 2E). The plasma membrane (PM) and cytoplasm (C) components were separated from S1; the former includes the plasma membrane, Golgi apparatus, endosomes, and lysosomes, as detected by flotillin1 (Fig. 2E). Tubulin confirmed that the C fraction consist of cytoplasm (Fig. 2E). A 1% portion of the cell homogenate (H) was loaded as a quantitation standard, PM and Mito from the 90% homogenate and C from the 1% homogenate were loaded for detection. Mitochondrial APP was almost equally distributed in the PM (1.03) and Mito fractions (1.18) in the control cells (Fig. 2E). However, in *app^Δ36^* cells, APP was approximately 1.6 times more abundant in PM (1.12) than in Mito (0.72) (Fig. 2E). These data indicated that deletion of the TM and JM region disturbed the localization of KPI-APPs in cells, especially their targeting to mitochondria.

### Morphological alteration of mitochondria in *app^Δ36^* cells

**Figure 3:**
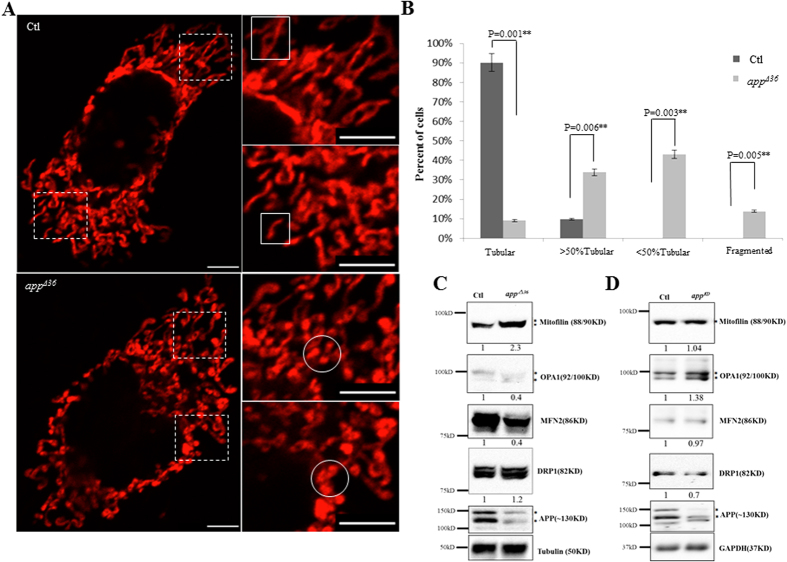
Mitochondrial morphology is altered in *app^Δ36^* cells. (**A**) Effect of mutant APP on mitochondrial morphology. Images were acquired with 100× oil-immersion objective. The images in the dotted box are shown at a higher resolution on the right (scale bar 10 μm). The normal tubular morphology of isolated mitochondria is indicated with the white box; spherical or elliptical mitochondrial with higher fluorescent intensity are indicated with circles. (**B**) Quantitative analysis of mitochondrial morphology. All images were acquired under same conditions without any processing. The mitochondrial morphology of 100 cells per genotype was scored visually from three independent experiments according to the indicated criteria. The aspect ratio (RA) of isolated tubular mitochondria was more than 2. A mitochondrial RA of less than 2 was considered to be spherical or elliptical mitochondrial fragments. The relative proportion of “tubular” and “spherical fragments” in one cell was the criterion for the mitochondrial morphology analysis. For the “tubular” group, only tubular mitochondria were found in the cells. “>50% tubular” indicated that more than half of the mitochondrial mass per cell were tubules; “<50% tubular” group indicated that the mass less than half tubules appeared, with at least 3 clearly tubular mitochondria; “fragmented” indicated cells containing spherical mitochondrial fragments with a maximum of 2 short tubules. Error bars represent the mean ± SEM. Statistically significant differences are indicated with asterisks (*P < 0.05 versus control, **P < 0.01 versus control). Mitochondrial fusion factors MFN2 and OPA1 are decreased in *app^Δ36^* cells (**C**) but not in *app*^*KD*^ cells (**D**). The immune-reactive proteins with multiple bands are indicated with asterisks.

As overexpression of APP_695_ in neuroblastoma cells results in a fragmented mitochondrial structure^16^, we initially investigated whether the 12 aa-deleted KPI-APPs impact mitochondrial morphology. Mito-DsRed was transfected into cells to visualize mitochondria by confocal microscopy. In normal cells, most mitochondria exhibited a tubular structure, as the aspect ratio (RA) of isolated mitochondria from mitochondrial network was more than 2. As shown in Fig. 3A, whole mitochondria in the control cells exhibited a normal tubular structure. Mitochondrial morphology in *app^Δ36^* cells differed from that in control cells (selected stable clones without *app* gene mutation and with normal APP expression after *app*-TALEN transfection). Spherical or elliptical mitochondria (an RA less than 2), with higher fluorescent intensity, appeared at the terminal of isolated tubular mitochondria (Fig. 3A).

To show the alteration in mitochondrial morphology more clearly, we quantitatively analyzed mitochondrial morphology. The relative proportion of “tubular” mitochondria and “spherical fragments” in one cell was the criterion for this analysis. We visually classified mitochondrial morphology into four groups, as refer the method in Song, Z.Y. *et al.*^21^. A total of 100 cells of each genotype were classified into “tubular”, “>50% tubular”, “<50% tubular” and “fragmented” groups. 90% of the control cells are classified into “tubular” group. The remaining 10% were classified into a “>50% tubular” group, as more than half of the mitochondrial mass per cell appeared to be tubular (Fig. 3B). In contrast, 9% of the *app^Δ36^* cells exhibited normal tubular mitochondria, and 34% of these cells were classified into the “>50% tubular” group and 43% into the “<50% tubular”, with less than half of the mitochondria appearing to be tubular. The other 14% of *app^Δ36^* cells were classified into the “fragmented” group (Fig. 3B). Bubbles appeared at the ends of tubular mitochondria in most *app^Δ36^* cells, indicating the swelling of these organelles (Fig. 3A). The quantitative data indicated that mitochondrial morphology was altered in *app^Δ36^* cells (Fig. 3B).

### Perturbed mitochondrial dynamics in *app^Δ36^* cells

Mitochondria are dynamic organelles that undergo continuous fusion and fission^22^, and alteration of mitochondrial morphology is always the result of a fusion/fission imbalance^23^. A number of proteins are involved in mitochondrial fission, including the mammalian homolog dynamin-related protein 1 (DRP1)^16^. However, no significant difference in the DRP1 protein level between the control and *app^Δ36^* cells was observed (Fig. 3C). Mitochondrial fusion is regulated through OPA1, Mfn1 and MFN2^24^, and we observed a decrease in MFN2 (0.4) and OPA1 (0.4) proteins in *app^Δ36^* cells (Fig. 3C). Crista remodeling is characterized by mitochondrial fragmentation^25^, and Mitofilin, a key factor in the regulation of crista morphology^26^ was increased (2.3) in *app^Δ36^* cells (Fig. 3C). These data indicate that alteration of mitochondrial morphology in *app^Δ36^* requires impairment of mitochondrial outer- and inner-membrane fusion induced through the down-regulation of MFN2 and OPA1.

To exclude the effect of APP expression in *app^Δ36^* cells, another mutant cell line, *app*^*KD*^ (a single clone cell line acquired by the same procedure as *app^Δ36^*), was evaluated for levels of mitochondrial fission and fusion proteins. Both the wild-type sequence and a 128-bp deletion in the coding sequence (CDS) are present in *app*^*KD*^, which exhibits decreased levels of APP (Fig. 3D). Compared with control cells, no significant difference in the mitochondrial fission factor DRP1 (0.7) and fusion factors MFN2 (0.97) and OPA1 (1.38) were found in *app*^*KD*^ cells (Fig. 3D). This finding indicated that the mitochondria morphology alteration in *app^Δ36^* cells was not due to the level of APP expression.

**Figure 4:**
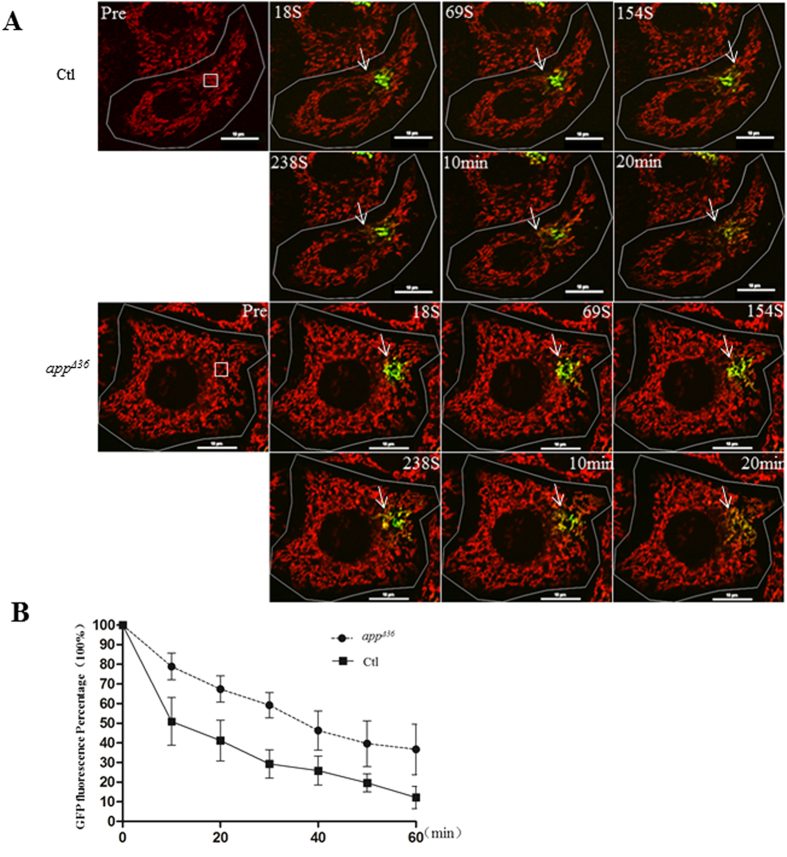
Mitochondrial dynamic perturbed in *app^Δ36^*. (**A**) The disperse of Mito-PA-GFP into the surrounding region in cells. Time-lapse imaging was performed with a 60× oil-immersion objective. After staining with Mito-tracker Red, Mito-PA-GFP expressed cells with dim fluorescence were randomly chosen. A 3 μm square was defined on the mitochondria as a region of interest (ROI) with 3× magnification in still frame (white square box). The PA-GFP fluorescence increased significantly when photo-activated with a 405 nm laser at an ROI for 1 s. The mitochondrial behavior of the entire cell was monitored every 10 s for 60 min. The disperse of Mito-PA-GFP through mitochondrial fusion can be observed by merging the green (Mito-PA-GFP) and red (Mito-tracker Red) frames. The yellow area (white arrow) increased over time (scale bar 10 μm). (**B**) Quantitative analysis of the Mito-PA-GFP disperses over time by normalization of the fluorescence intensity of individual mitochondria in an ROI. The GFP fluorescence percentage (the GFP fluorescence intensity of an ROI at different time points normalized by the highest intensity after activation) was averaged from 10 cells and error bars represent the mean ± SEM.

The decrease in mitochondrial fusion factors, MFN2 and OPA1, in *app^Δ36^* cells suggested the possible perturbation of fusion events. To measure the occurrence of mitochondrial fusion events, the methods mentioned by Wang, X. *et al.*^11^ and Song, Z.Y. *et al.*^26^ were applied. Cells were transfected with Mito-PA-GFP, which can be activated by a 405 nm laser with a significant increased fluorescence^27^. At 48 h after transfection, the cells were incubated with Mito-tracker Red, and mitochondria were tracked by time-lapse microscopy. A single Mito-PA-GFP-labeled cell was photo-activated at the defined region of interest (ROI), and its behavior was monitored every 10 s for 60 min. In cells with high mitochondrial fusion activity, the PA-GFP fluorescence intensity in the ROI was steadily diluted due to surrounding mitochondrial fusion, whereas PA-GFP fluorescence remained stable in cells with little or no mitochondrial fusion. Ten positively transfected cells were chosen randomly with same ROI defined. The mitochondria appeared yellow when the diluted Mito-PA-GFP signals and Mito-tracker Red signals were merged (Fig. 4A). Due to variable transfection efficiency of PA-GFP in different cells, the percentage of GFP fluorescence intensity in ROI (i.e., the GFP intensity at 10 min was normalized by the highest GFP intensity at 0 min) at each time point was then measured as an overall index for fusion events (Fig. 4B). At 10 min after activation, the control percentage was 51% compared to 79% in *app^Δ36^*; 42% in control ROI compared to 67% in *app^Δ36^* at 20 min was observed; 29% in control compared to 59% in *app^Δ36^* at 30 min. As shown in Fig. 4B, the GFP fluorescence faded faster in the control cells than in the *app^Δ36^* cells. These data suggested that mitochondrial fusion was slower in *app^Δ36^* cells compared with control cells.

### Impaired mitochondrial function in *app^Δ36^* cells

**Figure 5:**
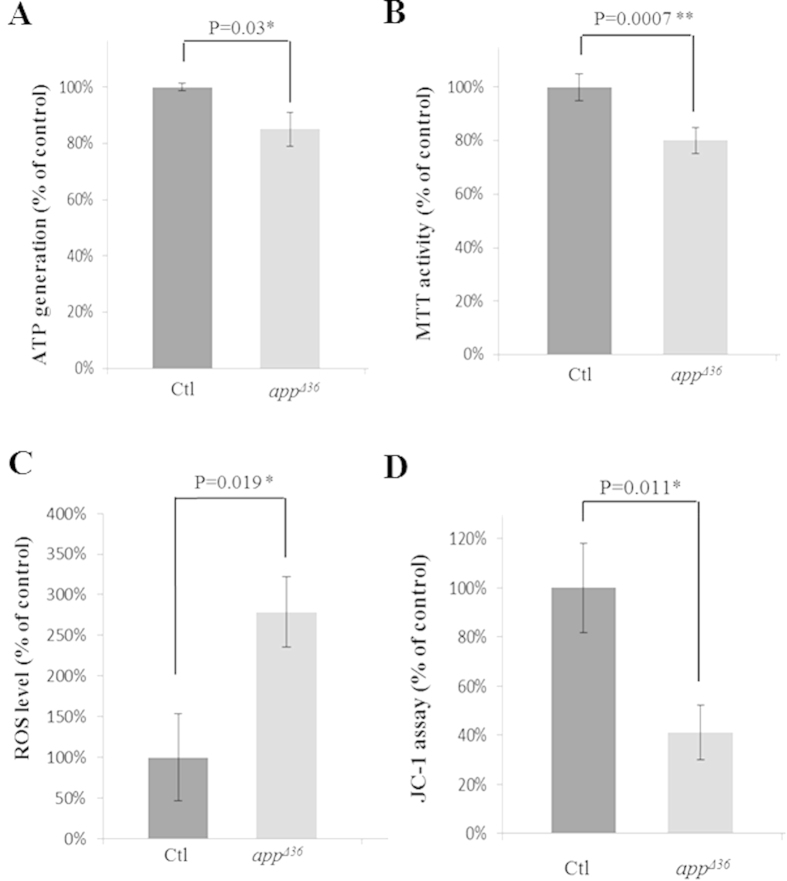
Mitochondrial function is impaired in *app^Δ36^* cells. The ATP generation (**A**), MTT activity (**B**), ROS levels (**C**) and the JC-1 assay (**D**) are shown. Error bar represents mean ± SD. Statistically significant differences are shown with asterisks (*P < 0.05 versus control, **P < 0.01 versus control).

The imbalance between fusion and fission alters mitochondrial morphology, which might impair mitochondrial function^14^,^28^. Considering that the morphological alteration of mitochondria in *app^Δ36^* might impair mitochondrial function, four parameters were measured: mitochondrial dehydrogenase activity was detected through an MTT [(3-(4, 5-dimethylthiazol-2-yl)-2, 5-diphenyltetrazolium bromide)] assay; the ROS level was assessed using a fluorescent probe (CM-H2DCFDA); ATP generation was estimated using a luciferase-based assay; and the mitochondrial membrane potential was measured using a JC-1 assay. A significant impairment in all four assessments was observed in *app^Δ36^* cells (Fig. 5A–D). ATP generation decreased by 15% compared with control cells (Fig. 5A). Mitochondrial dehydrogenase activity in *app^Δ36^* cells was 80% that of the control cells (Fig. 5B), and ROS generation was increased by 2.78-fold (Fig. 5C). In addition, the mitochondrial membrane potential was disrupted by approximately 59% (Fig. 5D). These data indicate the functional impairment of mitochondria, as well as alterations in mitochondrial morphology (Fig. 3A,B) in *app^Δ36^* cells.

## Discussion

TALEN is an effective tool for editing specific genomic regions. By fusing the *Fok I* nuclease with a specific TALEN, a targeted CDS can be cleaved to activate NHEJ repair, resulting in targeted modification such as insertions, deletions and substitutions. Most mutations are frame-shifts and may lead to gene knockout. In-frame mutants can also be generated, as in our study in which a specific domain was knocked, and in-frame insertions and substitutions in a targeted CDS can also be produced. These mutants are helpful for studying protein domain or amino acid specific functions. Although the mutations are randomly generated in a targeted region, the screening of a desired mutant is much easier compared with traditional forward genetic screening. Another approach for editing genomic-specific region is the CRISPR/Cas9 system.

More than half of the APP mutations found to be associated with familial forms of AD are located in the TM domain^29^. We generated the *app^Δ36^* cell line, which harbors KPI-APPs lacking a segment encompassing the TM region (719–723 aa) and C-terminal JM region (724–730 aa). The secondary structure of APP-TM, a straight α-helical conformation^4^, is highly flexible and sensitive to the surrounding lipid environment^4^,^30^; thus, loss of the TM region (719–723 aa) may lead to instability of APP at the membrane. It is known that the N-terminal JM region (687–700 aa) forms a hydrophobic cavity that facilitates a suitable TM region conformation^4^. We speculated that loss of the C-terminal JM region (724–730 aa) may also increase APP instability at the lipid bilayer. The TM domain is characterized as a dynamic structure, with conserved amino acid in diverse APP isoforms. These residues are critical for APP to adapt the fluctuations of the membrane thickness^4^. KPI-APPs lacking the TM-JM region should be less adaptive, which intends that only lipid bilayer with certain thickness is suitable for protein embedding. The family-AD mutations of APP-TM are usually point mutations with a single amino acid substitution. Although the impact of an amino acid substitution on secondary structure may be weak compared to the partial loss of the TM-JM region, as in *app^Δ36^*, the insertion of APP into the membrane could also be affected.

The cellular membrane system is crucial for protein sorting and localization, and following synthesis in the ER, APP is transported from the Golgi to the cell surface or the endosome^1^. The mitochondrial-targeting sequence directs APP to one of its final destination “mitochondria” via plasma membrane transporting. In neuron cells, APP_695_ was found be associated with mitochondrial translocation channels when ectopically overexpressed^11^. In addition, APP_695_ metabolism is altered due to mitochondrial accumulation in AD patients but not in age-matched controls^10^. We found that KPI-APPs could also be targeted to mitochondria, though the details of APP transport from ER to mitochondria is still obscure. In the normal human brain, APP is not present in mitochondria but in plasma membrane at higher levels^10^. The elevations in protein kinases accompany the mitochondrial accumulation of APP in the AD brain^10^. All these data suggest that the phosphorylated status of APP might be the key factor for mitochondrial localization. The lost JM region of APP in *app^Δ36^* is enriched of phosphorylated sites including “Y_728_”, “T_729_”, and “S_730_”. Thus, the decreased proportion of the mutant protein in the “Mito” fraction (Fig. 2E), indicates that the loss of phosphorylated residues in KPI-APPs might disrupt their post-translational modification in the cellular membrane system, resulting in reduced transport to mitochondria. Nonetheless, it remains to be determined whether the three phosphorylation sites individually or collectively influence APP mitochondrial localization. In addition, although the AD risk gene “*TOMM40*” is proven to function in APP mitochondrial localization^10^, no interaction between TOM40 and KPI-APPs can be detected (our unpublished data). Several experiments to elucidate the precise mechanisms involved in APP targeting to mitochondria are in progress, which might shed light on the mechanisms of mitochondrial abnormalities during AD pathogenesis.

Multiple proteolysis pathways exist for APP processing. APP can be cleaved directly by α/γ secretase in the “non-amylogenic” pathway at the cell surface or the trans-Golgi^2^. Cell membrane-localized APP is then re-internalized into endosomal compartment, which contains β/γ secretase, and generate Aβ in the “amyloid-genic” pathway^3^. However, the “amyloid-genic” pathway was not considered in the present study because β secretase (BACE1) is principally expressed in neurons^31^ , in contrast, HeLa cells possess only a modest capacity of Aβ production, with a low level of BACE1 protein level^32^, similar to AICD, a sequential β/γ secretase proteolytic product of APP. In addition, KPI-APPs have been demonstrated to be a substrate for mitochondrial γ secretase in non-neuron cells, and AICD is produced inside the mitochondria^20^. In *app^Δ36^* cells, α-secretase processing only occurred because the lost TM region contains the γ secretase site (Fig. 1C); thus, the proteolysis products are the large soluble N-terminal (sAPP_α_) and C-terminal APP (αCTF or C83) fragments. Mitochondrial AICD cannot be generated in the mutant.

Full length KPI-APPs are observed in outer mitochondrial membrane (OMM), and in association with translocase of the outer mitochondrial membrane 20 (TOM20)^20^. TOM20 is a receptor of the translocase of OMM complex (TOM complex) with TOM40 as the central channel^33^. The N-terminus of full length APP was inserted into the matrix, and its TM region was embedded into the OMM, with the C-terminus inserted into the inter membrane space. Mitochondrial C83 is generated by cytoplasmic enzymes with α-secretase activity, probably Omi^20^. In normal non-neuron cells, mitochondrial γ secretase cleaves C83 at the TM region to release AICD into the inter membrane space^20^. Although mitochondria are target for APP accumulation, the role of APP in mitochondria remains unclear^10^. Overexpressed KPI-APPs are associated with impairment of mitochondrial function^18^, and APP_695_ overexpression in neuronal cells results in mitochondrial fragmentation and abnormal distribution^11^. These data suggests that localization of APP in mitochondria is important for mitochondrial morphology and function. Our results show markedly altered mitochondrial morphology in cell with mutant APP (Fig. 3). To exclude the possibility that the alteration in mitochondrial morphology was due to the decreased mitochondrial localization of mutated APP, we examined an APP knockdown cell line that was acquired in the same way as the *app^Δ36^* line. We assumed that these APP knockdown cells (*app*^*KD*^) would exhibit decreased mitochondrial localization, though the cellular distribution of APP was as it is in normal cells. If the mitochondrial morphology alteration of the mutants is due to the APP expression level, the mitochondrial fusion factors OPA1 and MFN2 should also be decreased in APP knockdown cells. However, no change in OPA1 and MFN2 protein levels was found in the APP knockdown cell line (Fig. 3D). It is confirming that the alteration in mitochondria morphology in *app^Δ36^* was not caused by the level of APP expression. Mitochondrial dynamic analysis indicated that mitochondrial fusion was slowed, coincident with the decreases in OPA1 and MFN2 in *app^Δ36^* cells (Fig. 3E). Comparison of KPI-APPs proteolytic products between *app^Δ36^* and *app*^*KD*^ cells showed that the lost TM-JM region in *app^Δ36^* excluded mitochondrial KPI-APPs from γ secretase cleavage, such that mitochondrial AICD generation was blocked. Loss of mitochondrial AICD might affect the mitochondrial fusion process and alter mitochondrial morphology. A similar phenomenon was also found in stable APP_695_-overexpressing cell lines with significantly decreased OPA1^11^. We speculate that functional APP mitochondrial localization may be critical for mitochondrial morphology and function. In neuronal cells, APP_695_ is the main mitochondrial localized isoform^10^, and it might serve to ensure healthy mitochondria. In contrast, KPI-APPs may serve to protect mitochondria from dysfunction in non-neuron cells.

## Methods

### Cells and reagents

The HeLa cell line was purchased from China Center for Type Culture Collection (Wuhan). HeLa cells were grown in Dulbecco’s Modified Eagle’s medium (DMEM) (Hyclone, Thermo) supplemented with 10% fetal calf serum (Invitrogen, USA) at 37 °C in 5% CO_2_.

### Plasmid construction and transfection

The pTALE vectors, including pTALE-A, pTALE-T, pTALE-C, and pTALE-G, were purchased from Cwbiotech (Beijing), and digested using SpeI and HindIII or NheI and HindIII. Subsequently, the binding domains were ligated to the exon 17 of the *app* gene at the following sequence: upstream 5′-TGGTGATGCTGAAGAA-3′, downstream 5′-CCACCACACCATGATG-3′. For transient transfection, HeLa cells were seeded in 6-well plates followed by transfection with the verified constructs described above using Lipofectamine^TM^ 2000 Reagent (Invitrogen, USA). Genomic DNA was extracted from the transfected cells after 48 h and PCR was performed using an Eppendorf Mastercycler (Hamburg, Germany) with the following primers: 1 F 5′-TGACCTCCTTTCATTAGTATGATC-3′, 1R 5′-AATACCTTGAGCAGAATATTCAC-3′. Sequencing of the PCR product was performed to estimate TALEN efficiency.

### Clonal cell line isolation and genotyping

Cells were counted and serially diluted to a final concentration of 1 cell per 200 μl of medium in each well of 96-well plates. At least ten plates for each transfected population were plated. After 1 month, single clonal cell lines were expanded in 24-well plates for selection via sequencing and western blot analysis. Mutations at the TALEN target sites of selected cell lines were genotyped after sequencing the T/A clones of 1F/1R PCR products. At least 10 clones were sequenced for per cell line.

### RT-PCR and immunoblotting analysis

Total RNA was extracted using Trizol (Invitrogen, USA) according to the manufacturer’s instructions. Reverse transcription was performed using oligo-dT and MMLV (Promega, Madison, WI, USA). The *app* transcripts were amplified using the following primers:

2F 5′-GTTCTGGGTTGACAAATATCAAG-3′,

2R 5′-CTAGTTCTGCATCTGCTCAAAG -3′,

APP6/9U 5′-GGTGGTTCGAGTTCCTACA-3′,

APP9L 5′-TCGAGATACTTGTCAACGG-3′,

APP7U 5′-GCTGGTACTTTGATGTGACT-3′,

APP7/9L 5′-TGTTGTAGGAATGGCGCTG-3′,

APP8L 5′-GTTTAACAGGATCTCGGGC-3′,

GAPDH F 5′-ATCCCATCACCATCTTCCAG-3′,

GAPDH R 5′-CCATCACGCCACAGTTTCC-3′. The PCR products were separated on a 3% agarose gel and sequenced after purification.

Protein was extracted using General Protein Extraction Reagent (Bioteke, Beijing) and supplemented with protease inhibitor cocktail (Roche). A total of 10/20 μg protein was loaded and separated by 10% SDS-PAGE, followed by electrophoretic transfer onto 0.45-μm polyvinyl difluoride membranes. Antibodies against the following proteins were used: APP (Millipore 22C11 1:500; Sangon AB60097b at 1:1,000), DRP1 (Abcam, 1:5,000), OPA1 (Epitomics, 1:1,000), MFN2 (Proteintech, 1:2,000), Mitofilin (Proteintech, 1:2,000), Flotillin1 (Proteintech, 1:1,000), Tim23 (Proteintech, 1:1,000), GAPDH (Proteintech, 1:1,000) and β-tubulin (Abbkine, 1:5,000). The protein levels were quantified by scanning the blots using a ChemiDoc^TM^ XRS + imager (Bio-Rad, California, USA) and analysis with Image Lab^TM^ software, version 3.0 (Bio-Rad, California, USA).

### Cell fractionation

Nearly confluent cells were collected from 15 cm culture dishes after washing with PBS. The cells were collected by centrifugation at 1,000 rpm for 3 min. One-tenth of the cells was reserved for quantity control, and the remaining cells were suspended in mitochondria separation buffer (Beyotime, China) supplemented with a complete protease inhibitor cocktail (Roche Applied Science, USA). All the ensuing steps were carried out at 4 °C to avoid protein degradation. Cells were homogenized in a Dounce glass homogenizer (Botong tech., China) until >90% of the cells were positively stained with trypan blue. The cells were then centrifuged at 600 × g for 10 min; the supernatant was centrifuged at 1,000 × g for 10 min and then, 3,500 × g 10 min. The pellet (P1), considered as mitochondrial fraction, was re-suspended in mitochondria separation buffer for 2 times and centrifuged at 6,300 × g for 10 min. Then mitochondria were collected at 10,000 × g for 10 min. The supernatant (S1) was reserved for cytoplasm and plasm membrane separation. The S1 supernatant was further centrifuged at 6,300 × g and 10,000 × g for 10 min to pellet mitochondria. The “light membrane” fraction was prepared by centrifugation at 20,000 × g for 30 min, and the resulting pellet contained plasma membrane, Golgi apparatus, endosomes, and lysosomes referred as PM. The resulting supernatant S2 consisted of the cytoplasm and ER. This method refers to the previously published protocol of Pinton *et al.*^34^. All the cell fractions were used for immune-analysis.

### Immunostaining and imaging

Cells grown on slides were transfected with a plasmid expressing mitochondria-targeted Ds-Red. After incubating at 37 °C for 24 h, the cells were washed in pre-warmed PBS, and 4% paraformaldehyde was used to fix the cells for 15 min. The cells were washed three times with PBS and imaged. The mitochondrial images were acquired using the 100× oil-immersion objective with an A1R confocal microscope (Nikon, Japan). Mitochondria-targeted Ds-Red was excited by a 1-mW 561-nm laser, and images were acquired through a 595–650-nm filter. All images were acquired under the same conditions.

### Quantitative analysis of mitochondrial morphology

The aspect ratio (RA) of isolated mitochondria was the criterion for mitochondrial morphology. Mitochondrial with an RA value of more than 2 were considered to be tubular. Mitochondrial with an RA less than 2 were considered to be spherical fragments. The relative proportion of “tubular” and “spherical fragments” in one cell was the criterion for the mitochondrial morphology analysis. To determine morphology, the cells were visually classified into 4 morphological groups: “tubular”, “>50% tubular”, “<50% tubular” and “fragmented”. For the “tubular” group, only tubular mitochondria were found in the cell. “>50% tubular” indicated that more than half of the tubules appeared in mitochondrial mass per cell; “<50% tubular” indicated that less than half of the tubules appeared, with at least 3 clearly tubular mitochondria; “fragmented” indicated cells containing spherical mitochondrial fragments with a maximum of 2 short tubules. All the raw images used for mitochondrial morphology were analyzed without any processing. To avoid artificial results, the analysis was performed in a blind continuous fashion. The data of 100 cells from three independent experiments were analyzed per genotypes (control and mutant cells). This method refers to the previously published research of Song *et al.*^21^.

### Time-lapse imaging

Cells were seeded in glass-bottom dishes (Nest, China) and transfected with Mito-PA-GFP. At 48 h after transfection, the cells were incubated with Mito-tracker Red (Invitrogen, USA) and then placed in an incubation system (INU-TIZ-F1, Tokai hit, Japan) for microscopy (Nikon, Japan) with a controlled CO_2_ content, humidity and temperature. The live cells were track by microscopy using a 60× oil-immersion objective. Mito-PA-GFP expressing cells with dim fluorescence were randomly chosen; the fluorescence was regarded as background and corrected. A 3 μm square was defined on the mitochondria as a region of interest (ROI) in still frame. Fast and sufficient photo-activation of Mito-PA-GFP was achieved by exposing a 3× zoomed area to 1-mW 405-nm laser for 1 s, and the PA-GFP fluorescence increased significantly (100 times as mentioned in article of Song, Z.Y. *et al.*^26^). The entire cell image was acquired with a double-channel mode using an inverted laser-scanning confocal fluorescence microscope. Mito-tracker Red was excited by a 2-mW 561-nm laser, and images were acquired through a 595–650-nm filter. PA-GFP was imaged by excitation with a 1-mW 488-nm laser, and emission was recorded through a 525–550-nm filter. Images of mitochondrial behavior were acquired every 10 s for 60 min without phototoxicity or photobleaching. The GFP intensity at the ROI was measured synchronously using a Nikon ViewNX (Nikon, Japan). This method refers to the previously published research methods^11^,^27^.

### MTT assay

Cells were seeded into 96-well plates with 5,000 cells per well. After 24 h, each cell strain was added in three consecutive wells in 10 μl of MTT reagent (5 mg/ml, Beyotime, China) per well. The plate was incubated at 37 °C for 4 h. Subsequently, 100 μl of formazan reagent (Beyotime, China) was added per well, followed by 4 h of incubation until the purple formazan solution became soluble. The absorbance was measured at 570 nm using a multifunctional microplate reader (Infinite200 PRO, TECAN, Switzerland).

### ROS measurement

Cells were seeded into 6-well plates the day before. DCFH-DA (10 μM, Beyotime, China) was added to the plate at 1 ml per well after aspirating the medium. After incubation at 37 °C for 20 min, the cells were washed three times with DMEM. Subsequently, the cells were digested and ROS was measured under 488 nm excitation using a fluorescence microplate reader (Infinite200 PRO, TECAN, Switzerland).

### Assay for cellular ATP levels

ATP lysis buffer (Beyotime, China) was added to 6-well plates with 200 μl per well. The lysates were collected through centrifugation at 12,000 × g at 4 °C for 5 min. For the ATP assay, 100 μl of ATP testing reagent (Beyotime, China) per well was added to a 96-well plate containing 20 μl of supernatant. The plate was shaken and measured using a multifunctional microplate reader (Infinite200 PRO, TECAN, Switzerland). For the BCA assay, the supernatant samples were added to a 96-well plate after incubating with 200 μl of BCA reagent (Beyotime, China) at 37 °C for 30 min, and the absorbance was measured at 562 nm using a multifunctional microplate reader (Infinite200 PRO, TECAN, Switzerland).

### JC-1 assay

The positive control was treated with CCCP (10 mM) at 1:1,000 to medium at 37 °C for 20 min. The medium was aspirated, and 1 ml of medium and 1 ml of JC-1 working reagent (Beyotime, China) were added, followed by incubation at 37 °C for 20 min. Subsequently, the cells were washed with JC-1 staining buffer (Beyotime, China). An aliquot of 1 ml of medium was added to each well of the plate, and the absorbance was measured at 490 and 525 nm using a multifunctional microplate reader (Infinite200 PRO, TECAN, Switzerland).

### Statistical analysis

Statistical significance (P-value) was determined through a two-way analysis of variance and a t-test.

## Additional Information

**How to cite this article**: Wang, Y. *et al.* Lost region in amyloid precursor protein (APP) through TALEN-mediated genome editing alters mitochondrial morphology. *Sci. Rep.* **6**, 22244; doi: 10.1038/srep22244 (2016).
